# Pediatric Encephalopathy: Clinical, Biochemical and Cellular Insights into the Role of Gln52 of *GNAO1* and *GNAI1* for the Dominant Disease

**DOI:** 10.3390/cells10102749

**Published:** 2021-10-14

**Authors:** Gonzalo P. Solis, Tatyana V. Kozhanova, Alexey Koval, Svetlana S. Zhilina, Tatyana I. Mescheryakova, Aleksandr A. Abramov, Evgeny V. Ishmuratov, Ekaterina S. Bolshakova, Karina V. Osipova, Sergey O. Ayvazyan, Sébastien Lebon, Ilya V. Kanivets, Denis V. Pyankov, Sabina Troccaz, Denis N. Silachev, Nikolay N. Zavadenko, Andrey G. Prityko, Vladimir L. Katanaev

**Affiliations:** 1 Translational Research Center in Oncohaematology, Department of Cell Physiology and Metabolism, Faculty of Medicine, University of Geneva, CH-1211 Geneva, Switzerland; gonzalo.solis@unige.ch (G.P.S.); alexey.koval@unige.ch (A.K.); sabina.troccaz@unige.ch (S.T.); silachevdn@belozersky.msu.ru (D.N.S.); 2 St. Luka’s Clinical Research Center for Children, 119620 Moscow, Russia; vkozhanov@bk.ru (T.V.K.); szhylina@mail.ru (S.S.Z.); ivanovna-76@mail.ru (T.I.M.); arhelios@yandex.ru (A.A.A.); ei-doc@yandex.ru (E.V.I.); kate.bolshakova@gmail.com (E.S.B.); sagamonyanc@mail.ru (K.V.O.); soayvaz@gmail.com (S.O.A.); npc_prityko@mail.ru (A.G.P.); 3 Department of Neurology, Neurosurgery and Medical Genetics, Faculty of Pediatrics, Pirogov Russian National Research Medical University, 117997 Moscow, Russia; zavadenko@mail.ru; 4 Unit of Pediatric Neurology and Neurorehabilitation, Division of Pediatrics, Woman-Mother-Child Department, Lausanne University Hospital (CHUV), 1011 Lausanne, Switzerland; Sebastien.lebon@chuv.ch; 5 Center of Medical Genetics, Genomed Ltd., 115093 Moscow, Russia; dr.kanivets@genomed.ru (I.V.K.); dr.pyankov@genomed.ru (D.V.P.); 6 A.N. Belozersky Research Institute of Physico-Chemical Biology, Moscow State University, 119992 Moscow, Russia; 7 V.I. Kulakov National Medical Research Center of Obstetrics, Gynecology and Perinatology, 117997 Moscow, Russia; 8 School of Biomedicine, Far Eastern Federal University, 690090 Vladivostok, Russia

**Keywords:** pediatric encephalopathy, *GNAO1*, *GNAI1*, G proteins, dominant mutation, case report, molecular etiology, Gln52, GTP binding, protein–protein interactions, plasma membrane, Golgi

## Abstract

Heterotrimeric G proteins are immediate transducers of G protein-coupled receptors—the biggest receptor family in metazoans—and play innumerate functions in health and disease. A set of de novo point mutations in *GNAO1* and *GNAI1*, the genes encoding the α-subunits (Gαo and Gαi1, respectively) of the heterotrimeric G proteins, have been described to cause pediatric encephalopathies represented by epileptic seizures, movement disorders, developmental delay, intellectual disability, and signs of neurodegeneration. Among such mutations, the Gln52Pro substitutions have been previously identified in *GNAO1* and *GNAI1*. Here, we describe the case of an infant with another mutation in the same site, Gln52Arg. The patient manifested epileptic and movement disorders and a developmental delay, at the onset of 1.5 weeks after birth. We have analyzed biochemical and cellular properties of the three types of dominant pathogenic mutants in the Gln52 position described so far: Gαo[Gln52Pro], Gαi1[Gln52Pro], and the novel Gαo[Gln52Arg]. At the biochemical level, the three mutant proteins are deficient in binding and hydrolyzing GTP, which is the fundamental function of the healthy G proteins. At the cellular level, the mutants are defective in the interaction with partner proteins recognizing either the GDP-loaded or the GTP-loaded forms of Gαo. Further, of the two intracellular sites of Gαo localization, plasma membrane and Golgi, the former is strongly reduced for the mutant proteins. We conclude that the point mutations at Gln52 inactivate the Gαo and Gαi1 proteins leading to aberrant intracellular localization and partner protein interactions. These features likely lie at the core of the molecular etiology of pediatric encephalopathies associated with the codon 52 mutations in *GNAO1*/*GNAI1*.

## 1. Introduction

G protein-coupled receptors (GPCRs) represent the biggest receptor family in the animal kingdom [1]. The main intracellular GPCR effectors are heterotrimeric G proteins composed of the α, β, and γ subunits, of which the α-subunit is responsible for binding to guanine nucleotides. When bound to GDP, the G protein can exist as the heterotrimer and is competent to interact with the cognate GPCR. The activated receptor acts as a guanine nucleotide exchange factor, catalyzing the exchange of GDP for GTP on the Gα. This triggers dissociation of the G protein into Gα-GTP and the βγ-heterodimer, which can bind and activate downstream transducer proteins. When GTP on Gα is hydrolyzed, the inactive Gαβγ heterotrimer re-associates for a new cycle of activation by the GPCR. Alternatively, the Gα-subunit can be reloaded with GTP and continue its signaling activity [2].

Gα-subunits provide the main signal transduction specificity in GPCR-initiated signaling cascades; four main subgroups of Gα-subunits are identified: Gαs, Gαq, Gα12/13, and Gαi/o [3]. The latter group transduces the signals of a group of rhodopsin-like GPCRs including the opioid, α2-adrenergic, D2 dopaminergic, M2 muscarinic, and somatostatin receptors [4], and also receptors of the FZD family of GPCRs [5,6,7,8]. Of the Gαi/o proteins, Gαo is the major Gα-subunit of the nervous system across the animal kingdom [9,10], controlling both the development and adult physiology of the brain [11]. Gαo knockout (KO) mice showed a developmental delay during the first 3 weeks after birth, and a short half-life of only 7 weeks on average [12]. Gαo KO mice also presented multiple neurological abnormalities such as hyperalgesia, hyperactivity, generalized tremor with occasional seizures, and severe impairment of motor control [12,13].

Advances in the next-generation whole-exome sequencing have identified multiple de novo missense mutations in *GNAO1*—the gene encoding Gαo—as the cause of rare yet severe neurological syndromes ranging from developmental delay with various movement disorders to early onset developmental and epileptic encephalopathy (DEE) [14,15,16]. As epilepsy affects nearly 0.7% of the population with about one-third lacking effective treatment [17,18], the number of cases with de novo *GNAO1* mutations is expected to increase as sequencing of patients is further performed. The patients are heterozygous for *GNAO1* mutations. Heterozygous KO mice (*GNAO1^+/−^*) showed no movement disorders such as those seen in the homozygous loss-of-function animals [12]. Thus, the neurological disorders are caused by the dominant—and not simple loss-of-function—nature of *GNAO1* mutations in human patients [19]. Interestingly, all point mutations (>30) within the coding region of *GNAO1* correspond to highly conserved residues (e.g., identical between human, fruit fly and worm), indicating their involvement in basic Gαo functions [20]. Despite some insights into the potential mechanisms of pathological mutations [14,21], the molecular mechanisms of Gαo mutants driving to abnormal movement disorders and epilepsy still await the much-needed clarification to reveal potential therapeutic targeting approaches.

Similarly to Gαo, another Gαi/o protein, Gαi1 (encoded by the gene *GNAI1*, see Supplementary Figure S1 for the amino acid alignment showing high homology between the two Gα-subunits) shows prominent central nervous system expression; its genetic ablation reveals important neurological functions such as involvement in long-term potentiation [22]. In a further similarity to Gαo, de novo point mutations in *GNAI1*, some of them—in the same positions as those found in *GNAO1* mutant patients, have recently been described to cause dominant infantile neurological disorders with variable degrees of developmental delay, seizures, and hypotonia [23].

Among the amino acid found mutated both in *GNAO1* and *GNAI1* patients, de novo Gln52Pro substitutions have been described [23,24]. For the *GNAO1* Gln52Pro variant, the patient had DEE with normal brain MRI [24]. For the *GNAI1* Gln52Pro variant patient, DEE was similarly reported [23] (see Table 1). In our work, we here describe another Gln52 mutant *GNAO1* patient, harboring a hitherto unknown Gln52Arg substitution. We provide the clinical description along with the molecular investigation of the three Gln52 pathological mutations identified so far—Gαo[Gln52Pro], Gαi1[Gln52Pro], and the novel Gαo[Gln52Arg]. We show that, unlike other previously characterized encephalopathy variants, mutations in the Gln52 result in a loss of basic biochemical and cellular activities of the Gα-subunits, providing a novel basis for the molecular etiology of the *GNAO1*- and *GNAI1*-related pediatric encephalopathies.

## 2. Materials and Methods

### 2.1. Ethics Statement

A written informed consent was obtained by St. Luka’s Clinical Research Center for Children, (Moscow, Russia) from the parents for genetic testing and for publication of a case report.

### 2.2. Plasmids and Molecular Cloning

The plasmid for His_6_-RGS19 was previously described [2]. To produce recombinant encephalopathy mutants, the plasmids encoding His_6_-tagged human Gαo (isoform 1) and Gαi1 cloned in pET23b [2] were subjected to site-directed mutagenesis using the following primer pairs: Gαo-Q52P-for, 5′-GCACCATTGTGAAGCCGATGAAGATCATCCATGAAGATG-3′ and Gαo-Q52P-rev, 5′-GGATGATCTTCATCGGCTTCACAATGGTGCTTTTTCC-3′; Gαo-Q52R-for, 5′-GCACCATTGTGAAGCGGATGAAGATCATCCATGAAGATG-3′ and Gαo-Q52R-rev, 5′-GGATGATCTTCATCCGCTTCACAATGGTGCTTTTTCC-3′; and Gαi1-Q52P-for, 5′-GGATAATTTTCATCGGCTTCACAATTGTACTTTTACC-3′ and Gαi1-Q52P-rev, 5′-TACAATTGTGAAGCCGATGAAAATTATCCATGAAGCTGG-3′. The PCR products were treated with DpnI to remove the parental vector, transformed in the *E. coli* Top10 strain (Thermo Fisher Scientific, Waltham, MA, USA). The Gαo-GFP construct was cloned by inserting a GFP sequence between the Gαo residues Gly92 and Ile93 with a flexible linker (GGGGG) [25]. Briefly, Gαo sequences upstream and downstream of Gly92 as well as the GFP sequence were PCR amplified from the pcDNA3.1-Gαo (cDNA Resource Center, Bloomsberg, PA, USA) and pEGFP-C1 (Takara Bio, Kusatsu, Japan) plasmids using the following oligonucleotides: CMV-for, 5′-CGCAAATGGGCGGTAGGCGTG-3′ and GαoG62-rev, 5′-CCTCGCCCTTGCTCACGGGCCCGCCGCCACCTCCGCCCAAAGTGTCCATG-3′; GαoG62-for, 5′-GGACGAGCTGTACAAGGGCGGAGGCGGAGGTATCGAATATGGTGATAAGG-3′ and BGH-rev, 5′-GCAACTAGAAGGCACAGTCGAGG-3′; and GFP-for, 5′-GGAGGTGGCGGCGGGCCCGTGAGCAAGGGCGAGGAGCTGT and GFP-rev, 5′-ACCTCCGCCTCCGCCCTTGTACAGCTCGTCCATGCCGAGA-3′. The PCR products were then combined to generate via PCR the complete Gαo-GFP sequence, which was then cut with KpnI and NotI, and ligated into the same sites of the pEGFP-N1 plasmid (Takara Bio). The Q52P and Q52R mutants were generated by site-directed mutagenesis of the Gαo-GFP plasmid as described above. The mRFP-Gβ1 construct was created by exchanging the AgeI/BsrGImCerulean sequence from mCerulean-Gβ1 [26] with the corresponding sequence from pmRFP-C1 [27]. Similarly, the mRFP-Gγ3 was created by substituting the AgeI/BsrGI GFP sequence in the GFP-Gγ3 plasmid [27] with the mRFP sequence. Correctness of all resulting clones was analyzed by Sanger sequencing.

### 2.3. Expression and Purification of Gαo and Gαi1 Wild-Type and Mutant Proteins

Plasmids encoding 6xHis-tagged Gαo and Gαi1, cloned in pET23b for wild-type (WT), [Glu52Pro] and [Glu52Arg] mutants were transformed in the *E.coli* strain Rosetta Gami (λDE3). The cells were grown with shaking in LB medium at 37 °C until they reached OD_600_ = 1, then were cooled to 25 °C and induced with 0.5 mM IPTG. After 8–12 h of further growth, the cells were harvested, lysed in OneShot cell disrupter (Constant Systems, Northants, United Kingdom) and the proteins were purified from lysate supernatant using Ni-NTA affinity resin (Qiagen, Germantown, MD, USA). Purification was performed overall according to the manufacturer’s protocol using Tris-HCl buffer (pH 7.5) containing 150 mM NaCl. Additionally, after 2 washes with 100 bead volumes of indicated buffer, the beads were incubated overnight on a rotary shaker at 4 °C in the same buffer containing 0.1 mM DTT, 10% glycerol, 100 µM GDP, and 5 mM MgCl_2_ buffer. After this incubation, the beads were again washed 3× with 100× bead volumes of wash buffer and eluted with the same buffer supplemented with 0.3 M imidazole. Protein yields and purity after expression and purification were assessed by SDS-PAGE and Coomassie staining.

### 2.4. GTP Binding and Hydrolysis Assays

Recombinant human Gαo and Gαi1, wild-type or [Q52P] and [Q52R] mutants were brought into the 20 mM Tris-HCl buffer (pH 7.5) containing 150 mM NaCl by a 10,000-fold buffer exchange on Amicon 10K ultracentrifugation concentrators (Merck, Kenilworth, NJ USA). For measurement, 2 µM of indicated protein was incubated for 30 min in the black 384-well plate (Greiner, Kremsmünster, Austria) and then mixed with an identical volume of 2 µM BODIPY-FL-GTP or 2 µM BODIPY-FL-GTPγS (both from Thermo Fisher Scientific) in the buffer containing 20 mM Tris-HCl buffer (pH 7.5), 150 mM NaCl, 0.2% BSA and 10mM MgCl_2_ using the plate reader injector directly before measurement. The kinetics of in vitro G protein binding and/or hydrolysis was measured in the Infinite M200 Pro multiwell reader (Tecan, Männedorf, Switzerland) [28,29].

### 2.5. Antibodies and Reagents

Monoclonal antibody (mAb) against mRFP (Cat# sc-101526) was from Santa Cruz Biotechnology (Dallas, TX, USA), the mAb against His_6_ (Cat# 34650) from Qiagen, and the mAb against GM130 (Cat# 610822) was from BD Biosciences (Franklin Lakes, NJ, USA). Polyclonal antibody (pAb) against GFP (Cat# GTX113617) was from GeneTex (Irvine, CA, USA). Secondary Abs for Western blots (Cat# 115-035-062 and Cat# 111-035-144) and immunostaining (Cat# 115-165-146) were from Jackson ImmunoResearch (West Grove, PA, USA).

### 2.6. Cell Line and Culture Conditions

Male mouse neuroblastoma Neuro-2a (N2a; Cat# CCL-131 ATCC, Manassas, VA, USA) were maintained in MEM (Thermo Fisher Scientific), supplemented with 10% FCS, 2 mM L-glutamine, 1 mM pyruvate, and 1% penicillin-streptomycin at 37 °C and 5% CO_2_.

### 2.7. Co-Immunoprecipitation

The recombinant GST-tagged Nanobody against GFP [30] expressed in *E. coli* RosettaGami (λDE3, Merck) was purified with glutathione Sepharose 4B beads according to manufacturer’s instructions. Protein purity was assessed by SDS-PAGE and Coomassie blue staining. N2a cells were co-transfected with the different Gαo-GFP constructs and mRFP-Gβ1/mRFP-Gγ3 (1:1:1 plasmid ratio) or the His_6_-RGS19 construct (1:1 plasmid ratio). After 24 h of transfection, cells were resuspended with ice-cold GST-lysis buffer (20 mM Tris-HCl, pH 8.0, 1% Triton X-100 and 10% glycerol in PBS) supplemented with a protease inhibitor cocktail (Roche, Basel, Switzerland) and passed 10 times through a 25 G needle. Extracts were cleared by centrifugation at 15,000× *g* for 15 min at 4 °C, and supernatants were incubated with 2 µg of purified GST-tagged GFP-Nanobody for 30 min on ice. Then, 20 µL of Glutathione Sepharose 4B beads (GE Healthcare, Chicago, IL, USA) were added, and samples were rotated overnight at 4 °C. Beads were repeatedly washed with GST-lysis buffer, prepared for SDS-PAGE, and finally analyzed by Western blot using antibodies against GFP, mRFP, and/or His_6_-tag, as well as HRP-conjugated secondary antibodies for ECL detection. Quantification of blots was done using ImageJ from 5 independent experiments and statistical analysis was carried out using Student’s *t*-test.

### 2.8. Immunofluorescence and Microscopy

For microscopy, N2a cells were transfected for 6 h, trypsinized, and seeded on poly-L-lysine-coated coverslips in complete MEM for an additional 15 h before fixation. Cells were fixed for 20 min with 4% paraformaldehyde in PBS. For immunostaining, cells were permeabilized for 1 min using ice-cold PBS supplemented with 0.1% Triton X-100, blocked for 30 min with PBS supplemented with 1% BSA, incubated with the primary antibody in blocking buffer for 2 h at room temperature (RT), washed and subsequently incubated with Cy3-conjugated secondary antibody and DAPI in blocking buffer for 2 h at RT. Coverslips were finally mounted with Vectashield on microscope slides. Cells were recorded with a Plan-Apochromat 63×/1.4 oil objective on an LSM800 Confocal Microscope and further processed using the ZEN blue software (all Carl Zeiss, Oberkochen, Germany).

## 3. Results

### 3.1. Case Report: A Gln52Arg GNAO1 Pediatric Encephalopathy Patient

**Figure 1:**
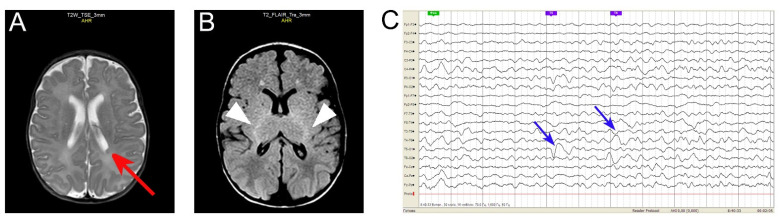
Brain MRI and EEG of the *GNAO1* Gln52Arg patient. (**A**,**B**) Brain MRI ((**A**): axial T2 weighted sequence, (**B**): axial FLAIR) reveal left posterior periventricular nodular heterotopia (PVNH, arrow in (**A**)) and hyperintensity of the posterior limb internal capsulae (arrowheads in (**B**)). (**C**) EEG reveals left posterior temporoparietal slowdowns (arrows).

This male Russian infant (currently 2 years old) displayed severe, early-onset developmental and epileptic encephalopathy (see Figure 1, Supplementary Table S1). Following the informed consent, whole-exome sequencing revealed a hitherto undescribed Gln52Arg (c.155A > G) heterozygous mutation in exon 2 of *GNAO1*. Predictive algorithms following ACMG criteria [31] characterize the mutation as likely pathogenic; no other pathogenic mutations were identified (data not shown). Sanger sequencing confirms the mutation (Supplementary Figure S2A). As both parents do not harbor the mutation (Supplementary Figure S2B,C), the *GNAO1*c.155A > G (Gln52Arg) is concluded to be a de novo mutation. The clinical manifestations of this hitherto undescribed de novo Gln52Arg mutation in *GNAO1* are reminiscent of the previously observed Gln52Pro mutations in *GNAO1* and in *GNAI1* [23,24]. Table 1 provides a comparison of the clinical features of the three patients with mutation in the codon Gln52.

### 3.2. Biochemical Characterization: Gαo[Gln52Pro], Gαi1[Gln52Pro], and the Novel Gαo[Gln52Arg] Mutants Are Devoid of the Basal GTP Binding Activity

In order to study the properties of the Gαo/i1Gln52 mutant proteins, we performed site-directed mutagenesis and cloned the Gαo[Gln52Pro], Gαi1[Gln52Pro], and the novel Gαo[Gln52Arg] mutant proteins for bacterial and mammalian cell expression (see Section 2). 

**Figure 2:**
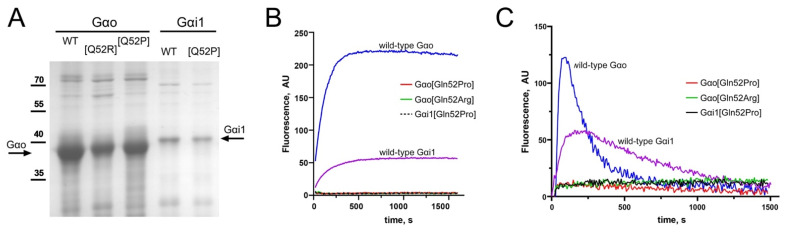
The Gln52 mutant Gαo/Gαi1 proteins are deficient in GTP binding and hydrolysis. (**A**) Coomassie-stained gel shows protein yields after purification of Gαo/Gαi1 recombinant proteins (arrows); expression and purification were performed in parallel. A decrease in the yields of mutant proteins (Q52P and Q52R) as compared to the wild-type (WT) versions can be seen as indicative of the overall lesser stability of the mutants. (**B**,**C**) Time course of binding of 1 μM BODIPY-GTPγS (**B**) or BODIPY-GTP (**C**) and 1 μM wild-type or mutant Gαo/Gαi1 proteins. While the wild-type proteins display the characteristic binding (**B**) and binding-hydrolysis (**C**) curves, the three mutant versions are incompetent in binding and hydrolysis of GTP). The gel (**A**) and the GTP binding (**B**) and binding-hydrolysis (**C**) experiments are representatives of three independent experiments.

Upon recombinant expression in and purification from *E. coli*, the three mutant proteins were well-expressed in a soluble form, although their expression levels were reduced as compared to the wild-type Gαo and Gαi1 (Figure 2A). This pattern could indicate that the mutant protein stability is somewhat—but not dramatically—reduced.

In order to characterize the fundamental biochemical property of the G proteins—their ability to bind GTP—we first utilized the non-hydrolyzable fluorescent analog of the guanine nucleotide, BODIPY-GTPγS, which increases its fluorescence upon the uptake by a G protein [2,27,28,32,33,34] (Figure 2B). While the wild-type forms of Gαo and Gαi1 robustly bind BODIPY-GTPγS (although with unequal kinetics and quantum yield, which is expected as the two proteins are non-identical), the three Gln52 mutant Gαo/i1 forms display a complete inability to bind the guanine nucleotide (Figure 2B).

We further tested a hydrolyzable fluorescent GTP analog, BODIPY-GTP, whose interaction with an active G protein is seen as a transient rise in fluorescence (indicative of the nucleotide binding) followed by a decay in fluorescence (indicative of GTP hydrolysis due to the lower quantum yield the resultant fluorophore on the protein) [2,28,33,35] (Figure 2C). Comparative analysis of the BODIPY-GTPγS and BODIPY-GTP curves permits assessment of the binding and hydrolysis rates, providing the biochemical fingerprint of a G protein [2]; it can be seen that both binding and hydrolysis of GTP by Gαo is faster than by Gαi1 (Figure 2B,C), in agreement with prior observations [2,33]. It can also be seen that the three mutant proteins are as incapable of interacting with BODIPY-GTP as they are with the non-hydrolyzable GTP analog (Figure 2B,C).

Thus, we conclude that the three clinically observed pediatric encephalopathy Gαo/Gαi1 mutations in the position Gln52, Gαo[Gln52Pro], Gαi1[Gln52Pro], and Gαo[Gln52Arg], are all devoid of the basal GTP binding and hydrolysis activity. This deficiency observed for the recombinantly produced proteins must be reflected by deficient target protein interactions in cells, as confirmed in the next section.

### 3.3. Cellular Characterization: Gln52 Mutant Proteins Are Deficient in Interaction with Gαo Partner Proteins

**Figure 3:**
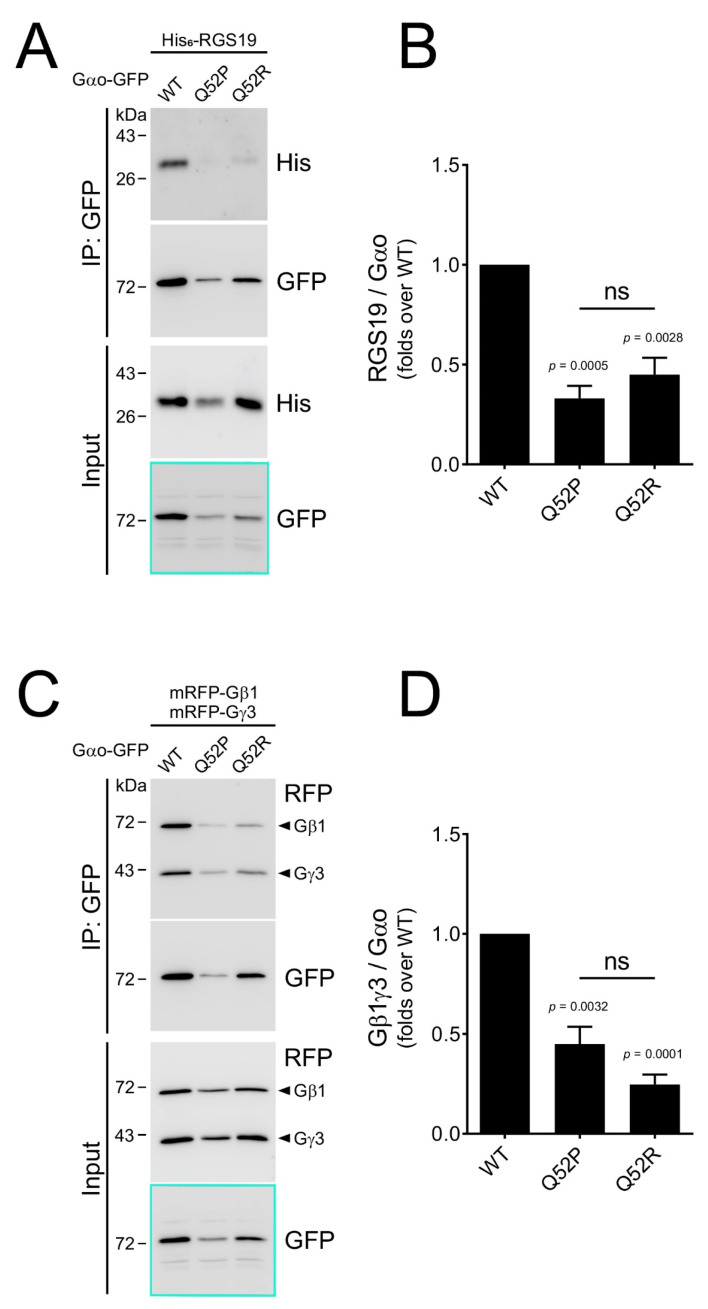
Pediatric encephalopathy mutations in Gln52 of Gαo strongly impair the G protein interaction with its partners in N2a cells. (**A**,**B**) Gαo-GFP wild-type (WT) and its Q52P and Q52R mutants were expressed in N2a cells together with His6-RGS19. The Gαo-GFP constructs were immunoprecipitated (IP) and the co-precipitation of RGS19 was analyzed by Western blotting (**A**). Note that the expression of both Gln52 mutants is reduced compared to Gαo WT (cyan box in the Input, also seen in panel (**C**). Quantification of the co-precipitation of RGS19 (**B**) normalized to the precipitated Gαo. Data shown as the mean ± SEM from 5 independent experiments. (**C**,**D**) Gαo-GFP wild-type and its Q52P and Q52R mutants were expressed in N2a cells together with mRFP-Gβ1 and mRFP-Gγ3. The Gαo-GFP constructs were immunoprecipitated (IP) and the co-precipitation of the Gβ1γ3 heterodimer was analyzed by Western blotting (arrowheads, (**C**)). Quantification of the co-precipitation of Gβ1γ3 (**D**) normalized to the precipitated Gαo as in (**B**). P-values for the statistical significance of differences to the wild-type protein are shown in (**B**,**D**); the differences between the two mutant proteins are insignificant (ns).

In order to test whether the Gln52Pro and Gln52Arg Gαo mutants interact with their targets in neuronal cells, we expressed them, along with the wild-type protein, in the GFP-tagged forms, in the N2a neuroblastoma cells [27]. Noteworthy, in these tagged forms, the GFP sequence is inserted internally, between the Gαo residues Gly92 and Ile93 (see Section 2), preserving the normal post-translational modifications, partner protein interactions, intracellular localization, and functioning of Gαo [27]. In agreement with the reduced yet robust expression in bacteria (Figure 2A), we see that the levels of Gαo[Gln52Pro] and Gαo[Gln52Arg] upon expression in N2a cells are reduced as compared to the wild-type protein (see cyan-highlighted boxes in Figure 3A,C).

To investigate how well the mutant variants interact with the Gαo partners, we co-expressed Gαo along with two types of partner proteins, which was followed by the co-IP experiments. The first partner we tested was RGS19, which interacts preferentially with the GTP-loaded form of Gαo [2]. We argued that if the Gαo[Gln52] mutant variants are unable to bind GTP (Figure 2B,C), they should display a reduced interaction with RGS19 in the cellular setting. This is indeed what we find: even upon correction for the reduced expression levels, Gαo[Gln52Pro] and Gαo[Gln52Arg] display a 2-to-3-fold reduced capacity to interact with RGS19 in N2a cells (Figure 3A,B). These findings agree well with the biochemical observations (Figure 2) and suggest that both in vitro and in vivo, the mutant proteins are impaired in the GTP binding.

The second interaction partner we chose to test was the Gβγ heterodimer, interacting with Gαo in the GDP-loaded form of the G protein [36]. Interestingly, this interaction was also found reduced 2–4-fold upon the Gln52 mutation in Gαo (Figure 3C,D), which may suggest that the ability of Gαo to bind GDP (or to adopt the proper conformation upon the interaction with the nucleotide) is aberrated in Gαo[Gln52Pro] and Gαo[Gln52Arg].

### 3.4. Subcellular Localization: Severe Loss of Plasma Membrane but Not Golgi Staining by the Gln52 Mutant Proteins

Gαo displays a conservative dual localization in different cell types including neuronal cells: plasma membrane and Golgi [27]. Furthermore, while at the plasma membrane Gαo naturally colocalizes with the Gβγ heterodimer, its localization and functioning at Golgi is independent of Gβγ [27]. We thus questioned whether the decreased cellular interaction with Gβγ we observe for the Gln52 Gαo mutants (Figure 3) may be linked with the changed intracellular localization of the mutant proteins. 

**Figure 4:**
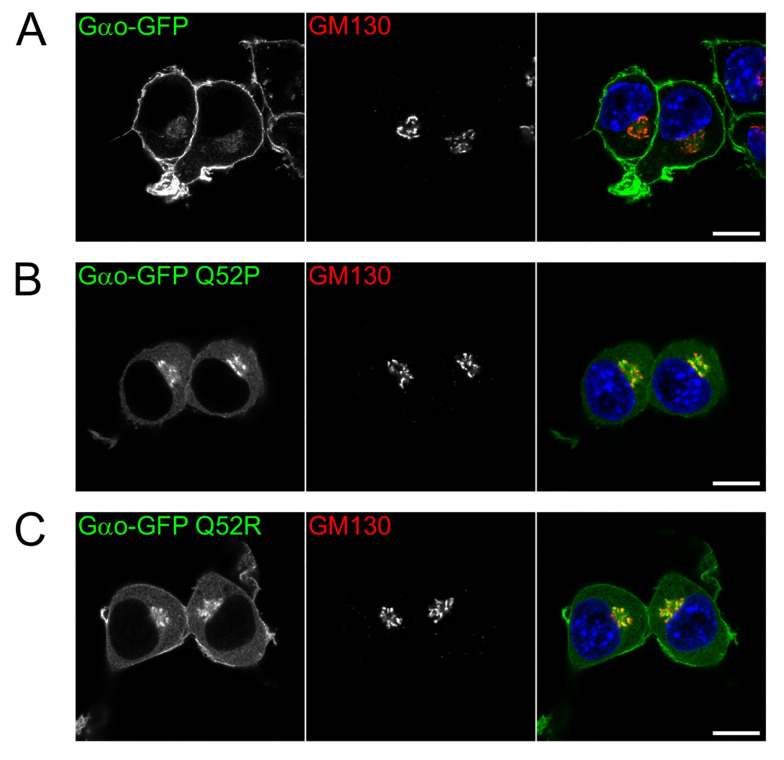
Pediatric encephalopathy mutations in Gln52 of Gαo strongly impair its plasma membrane (PM) localization. Representative confocal images of N2a cells expressing Gαo-GFP wild-type (**A**) and its Q52P (**B**) and Q52R (**C**) mutants. Cells were immunostained against GM130 as a Golgi marker; DAPI stained the nuclei in blue. Note that both Gln52 mutants showed a strong reduction in PM association and seemed to accumulate at the Golgi region and cytosol instead. Scale bars, 10 µm.

To address this issue, the intracellular localization of the internally GFP-tagged versions of Gαo (wild-type and the two mutant forms) was compared in the N2a cells (Figure 4). While the wild-type Gαo depicts the standard dual localization, at the plasma membrane and Golgi [27] (colocalizing there with the Golgi marker GM130, Figure 4A), the intracellular localization of Gαo[Gln52Pro] and Gαo[Gln52Arg] was perturbed. Specifically, both mutants show a dramatically decreased plasma membrane localization (the proline substitution is even stronger than the arginine one, Figure 4B,C). Reciprocally, the cytosolic content of the mutant proteins is clearly increased. Curiously, unlike the plasma membrane localization, the Golgi localization of the two mutants is not reduced but even increased as compared to the wild-type Gαo localization pattern (Figure 4). We thus conclude that the encephalopathy Gln52 mutations lead to a drastic reduction of the Gαo localization to one of the two major localization and signaling sites—the plasma membrane.

## 4. Discussion

More than 30 point mutations in *GNAO1* have been described to underline different manifestations of pediatric encephalopathy: epileptic seizures, motor dysfunctions, or the combination of both, accompanied by a developmental delay [14,15,16,37]. These clinical features place *GNAO1*-encephalopathy into the bigger group of developmental and epileptic encephalopathies [38,39]. The number of *GNAO1*-encephalopathy cases increases steadily since 2013, and it is clear that this rare neurological disease currently suffers from underdiagnosis due to the inaccessibility of whole-genome/exome or even targeted sequencing to many pediatric centers worldwide.

Recently, a number of mutations in a related gene *GNAI1*, many of them identical to those in the *GNAO1*-encephalopathy (Supplementary Figure S1), have been described to cause similar clinical manifestations in children [23]. Among the amino acids found mutated both in the *GNAO1*- and *GNAI1*-encephalopathy, the Gln52Pro mutations have been identified [23,24]. Here, we describe a hitherto unknown Gln→Arg mutation in the same site of *GNAO1*. The infant suffers from a combination of developmental delay, hypotonia, and seizures, accompanied by mild structural brain abnormalities (Figure 1). Although epileptiform discharges may be due to the periventricular nodular heterotopia (PVNH), the severity of the developmental outcome of our patient cannot be explained by this focal neuronal migration defect alone and is rather expected to result from an abnormal brain development related to the *GNAO1* variant. Brain MRIs are usually normal in *GNAO1*-related encephalopathies, and the discovery of a PVNH might be coincidental (although we cannot exclude a possible link with the genetic variant); that needs to be confirmed with further studies.

Despite some insights [14,21], the molecular mechanisms of the Gαo/Gαi1 mutants driving the pediatric encephalopathies still remain unclear. A basic feature of a G protein is its ability to bind and hydrolyze GTP. We here have assessed this capacity of the three described mutants: Gαo[Gln52Pro], Gαi1[Gln52Pro], and the novel Gαo[Gln52Arg] finding that all three mutations in the Gln52 site result in the incompetence of the mutant protein to bind and hydrolyze GTP. Resultingly, upon expression in a neuronal cell line, the mutant proteins interact poorly with RGS19—a partner of Gαo/i recognizing the GTP-loaded conformation of the G protein. Interestingly, when assessing the interaction with the Gβγ heterodimer, the partner interacting with the GDP-form of Gαo/i, the Gln52 mutants also display a strongly decreased interaction. 

These findings jointly indicate that the pathological mutations in the Gln52 site of *GNAO1* and *GNAI1* result in the mutant Gαo/Gαi1 proteins deficient in holding any guanine nucleotide (GTP or GDP). Alternatively, our data may be interpreted by the inability to bind GTP, along with the improper conformation adopted in the GDP-binding state not permitting efficient interaction with Gβγ. The reduced interaction with Gβγ upon Gln52 mutations is paralleled by the loss of Gαo from one of its two major subcellular localization and signaling zones—the plasma membrane. It can be hypothesized that the reduced ability to bind GDP or the inability to adopt the proper conformation in the GDP-binding state, leading to the loss of interaction with Gβγ, is the cause of loss of the mutant Gαo from the plasma membrane (and concomitant increase in the cytoplasm). Indeed, the strong lipidation-mediated anchoring of Gβγ at the plasma membrane is an important factor in keeping Gα-subunits at this location [40,41,42]. In contrast, with the localization of Gαo at the Golgi being Gβγ-independent [27], the Gln52 Gαo mutations do not display a reduction in the Golgi localization (rather an increase as compared to the wild-type protein, Figure 4). The subcellular localization of three other Gαo pathological point mutants (Gly203Arg, Ile279Asn, and Asp174Gly) has been previously analyzed and found not to affect the plasma membrane localization [14], in contrast to what we see for the Gln52 mutants (Figure 4). It is worth noting that the retained Golgi localization of the Gln52 mutants is indicative of the proper lipid modifications of the Gln52 Gαo mutants [43].

Thus, a model emerges whereas the encephalopathy Gln52 mutations in Gαo/Gαi1 proteins lead to their inability/reduced ability to interact with guanine nucleotides, leading to reduced interactions with the key partner proteins and loss of the mutant proteins from one of the two major subcellular localization and signaling sites—the plasma membrane. The resulting cellular consequences must be devastating. Given the key role of Gαo/Gαi1 in signaling by a multitude of neuronal GPCRs [4,44], both the developmental and the adult physiology stages of the central nervous system are expected to be affected by the mutations, ultimately resulting in the observed clinical manifestations. 

We wish to underline that the incompetence to interact with guanine nucleotides we ascribe to the Gln52 mutations in *GNAO1* and *GNAI1* is unlikely to be the general molecular feature for all the >30 point mutations identified in either gene in the pediatric encephalopathy patients. Indeed, we have explicitly shown that many encephalopathy mutants of Gαo are competent to uptake GTP in the BODIPY-GTPγS experiments ([45] and our unpublished observations), just as it has been shown by others for the Gαo[Arg209His] mutant [46] or for Gαi1[Glu245Lys] (corresponding to Glu246 in Gαo, see Supplementary Figure S1) [47]. While the feature of differential effects of different encephalopathy mutations on the GTP binding and hydrolysis needs a dedicated and separate investigation, we conclude that some of the encephalopathy mutants, represented by the Gln52 mutations in Gαo and Gαi1 we described here, are deficient in these basic features of a G protein, and we further suggest that this basic biochemical deficiency and the devastating cellular outcomes of it are at the core of the dominant nature of these particular heterozygous mutations causing pediatric encephalopathy.

We think that the insights we provide will be instrumental for the eventual development of molecular therapy to treat this devastating pediatric neurological disorder.

## Figures and Tables

**Table 1:** Description of patients with *GNAO1* and *GNAI1* Gln52 variants.

Patient Characteristics	*GNAO1* Gln52Pro *	*GNAI1* Gln52Pro	*GNAO1* Gln52Arg
Gender	not reported	Male	Male
Mutation	c.155A > C	c.155A > C	c.155A > G
Inheritance	de novo	de novo	de novo
Neurodevelopment and neurological features	not reported	Severe intellectual disability, autism spectrum disorder, hypotonia, lower limb hypertonia	Severe developmental delay **, dystonia in limbs
Extra neurological findings	not reported	Mild tricuspid regurgitation, severe constipation, asthma	Sleep disorders
Epilepsy	Onset <3 years, spasms	Onset age 6 years,	Onset age 1.5 weeks, focal spasms
EEG	Multifocal epileptiform discharges with slow background activity	Focal right posterior slowing	Multifocal epileptic activity (1 month)
Anti-seizure medication	not reported	Valproic acid	Valproic acid and levetiracetam.
MRI	Normal	Enlarged pericerebral spaces. Fronto-temporal atrophy (1 year), persistent at 3 years	Enlarged pericerebral spaces and ventricles. Left posterior periventricular nodular heterotopia (PVNH). PLIC (posterior limb internal capsulae) hyperintensity (38 days). Immature myelinization (32 months)
Reference	Rim et al., 2018 [24] *	Muir et al., 2021 [23]	This work

* the clinical description of the *GNAO1* Gln52Pro patient available in [24] is scarce. ** the current age of the patient (2 years) precludes proper assessment of any intellectual disabilities.

## Data Availability

The data presented in this study are fully disclosed in the main article and its supplementary materials.

## Supplementary Materials

The following are available online at https://www.mdpi.com/article/10.3390/cells10102749/s1, Table S1, Description of the patient with the *GNAO1* Gln52Arg variant, age progression; Figure S1, Alegnment of the amino acid sequences of Gαo and Gαi1; Figure S2, Sanger sequencing of the pediatric encephalopathy patient and his parents.
