# Supplementary material for: Pediatric Encephalopathy: Clinical, Biochemical and Cellular Insights into the Role of Gln52 of GNAO1 and GNAI1 for the Dominant Disease
Source: Cells. 2021 Oct 14;10(10):2749. doi: 10.3390/cells10102749 (PMC8535069; doi:10.3390/cells10102749)
Supplement: Supplementary file 1 [file cells-10-02749-s001.zip › cells-1339625-supplementary.pdf]

Pediatric encephalopathy: clinical, biochemical and cellular insights into the role of Gln52 of *GNAO1* and *GNAI1* for the dominant disease

Supplementary Materials for the article contain: Supplementary Table S1, Supplementary Figures S1 and S2.

Supplementary Table S1. Description of the patient with the *GNAO1* Gln52Arg variant, age progression.

	1.5 weeks-2 months	8 months	32 months
Neurological examination/ development	Axial hypotonia	Severe developmental delay with axial hypotonia, poor eye contact	Severe developmental delay, sleep disturbance
Seizures	Focal , facial clonia and hyperemia and tonic	Focal seizure: starring and loss of contact	Epileptic spasms
EEG	Multifocal epileptiform activity	Background rhythm slow down	Multifocal epileptiform activity
Involuntary Movement	Dystonia in limbs	Dystonia, stereotypia	Dystonia, bruxism, spasm. Abnormal motor skills
Seizure control	-	Refractory	-
Anti-seizure medication	Valproic acid, diazepam. Dexamethasone to suppress burst patterns as seen on EEG	Valproic acid and levetiracetam	Valproic acid and levetiracetam.
Brain MRI	Enlarged subarachnoid spaces and ventricles. Left posterior periventricular nodular heterotopia (PVNH). Hypersignal of PLICs		PVNH, Myelination delay

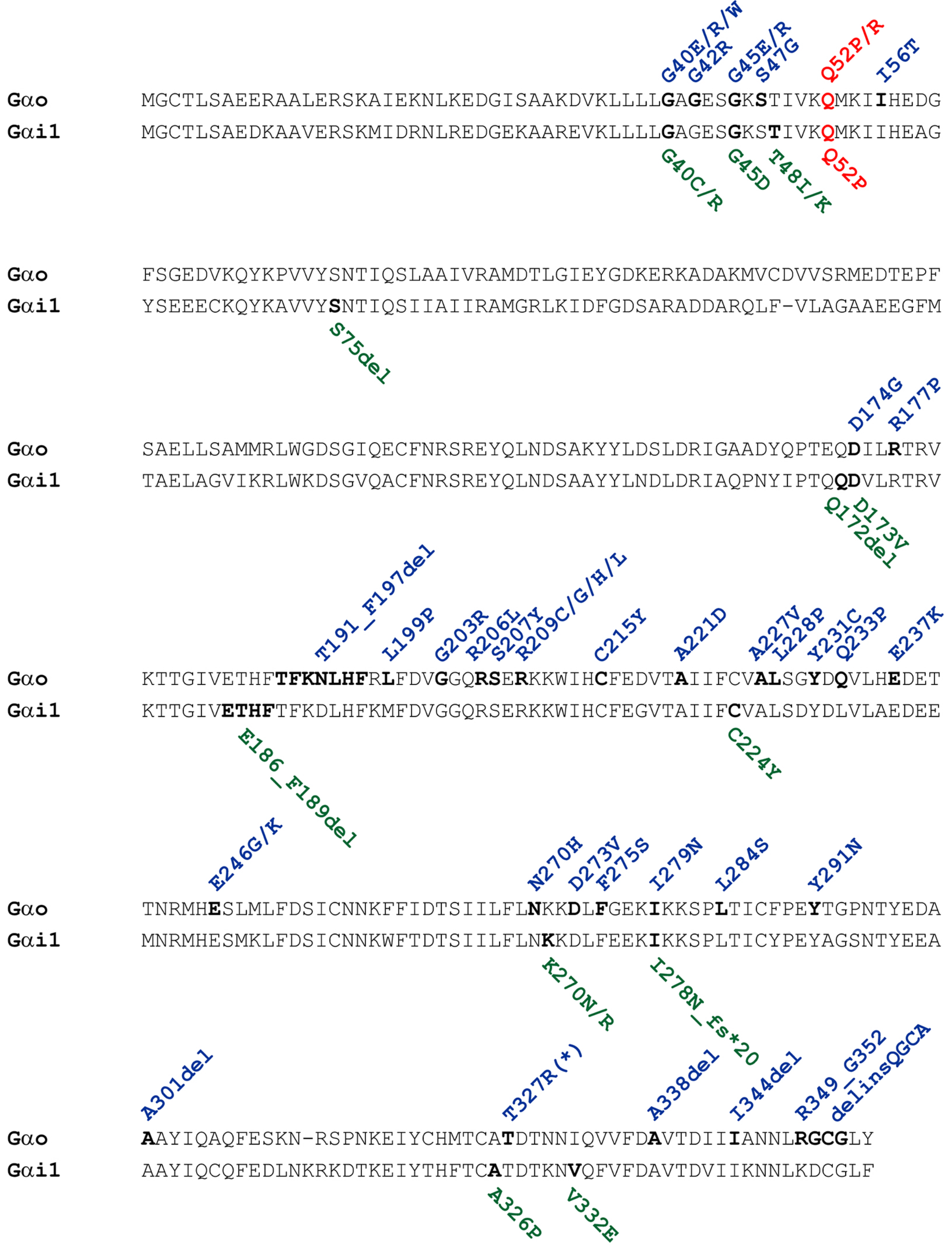

Supplementary Figure S1. Alignment of the amino acid sequences of Gαo (isoform 1) and Gαi1. Mutations reported in *GNAO1*/*GNAI1* pediatric encephalopathies are shown (the T327R mutation marked with a star causes a speech disorder but not encephalopathy). The mutations in the position Gln52 studied in this work are shown in red.

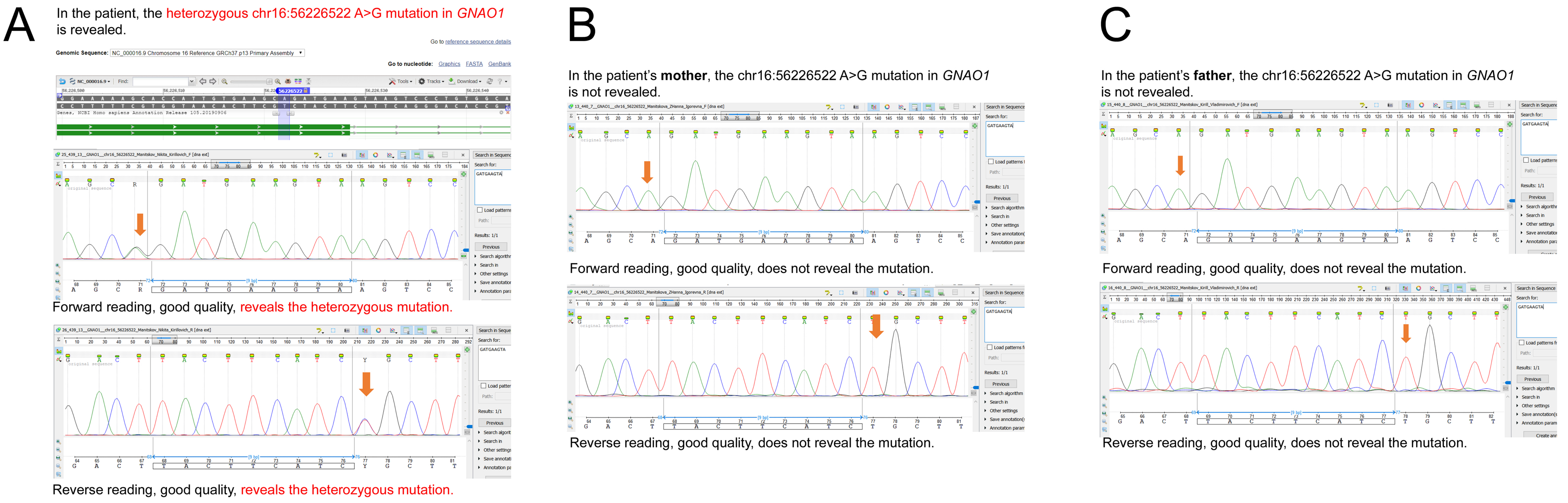

Supplementary Figure S2. Sanger sequencing of the pediatric encephalopathy patient and his parent reveals a heterozygous chr16:56226522 A>G mutation in *GNAO1* (A), absent in his monther (B) and father (C). Each sequencing is presented with a forward (upper panels) and reverse (lower panels) readings. Note that no signs of mosaicism in the chr16:56226522 A>G mutation can be seen in the electropherogram in (A).
